# Aspiration sclerotherapy combined with pasireotide to improve reduction of large symptomatic hepatic cysts (SCLEROCYST): study protocol for a randomized controlled trial

**DOI:** 10.1186/s13063-015-0607-3

**Published:** 2015-03-07

**Authors:** Titus FM Wijnands, Tom JG Gevers, Leo J Schultze Kool, Joost PH Drenth

**Affiliations:** Department of Gastroenterology and Hepatology, Radboud University Medical Center, PO Box 9101, 6500 HB Nijmegen, The Netherlands; Department of Radiology, Radboud University Medical Center, PO Box 9101, 6500 HB Nijmegen, The Netherlands

**Keywords:** Hepatic cyst, Polycystic liver disease, Aspiration sclerotherapy, Somatostatin analogues, Pasireotide

## Abstract

**Background:**

Aspiration sclerotherapy is an effective therapeutic option for large symptomatic hepatic cysts. However, incomplete cyst reduction following aspiration sclerotherapy is frequently reported. Strong post-procedural cyst fluid secretion by cholangiocytes, which line the epithelium of the hepatic cyst, seems to be associated with lower reduction rates. Previous studies showed that somatostatin analogues curtail hepatic cyst fluid production. This trial will evaluate the effect of aspiration sclerotherapy combined with the somatostatin analogue pasireotide on cyst reduction. By combining treatment modalities we aim to improve cyst reduction leading to greater symptomatic relief and reduced rates of cyst recurrence.

**Methods/Design:**

This single center, randomized, double-blind, placebo-controlled clinical trial evaluates the additional effect of pasireotide when combined with aspiration sclerotherapy in patients with a large (>5 cm) symptomatic hepatic cyst. A total of 34 participants will be randomized in a 1:1 ratio. In the active arm, patients will receive pasireotide (long-acting release, 60 mg injection) two weeks prior to and two weeks following aspiration sclerotherapy. Patients in the control arm will receive placebo injections at corresponding intervals. The primary outcome is proportional cyst diameter reduction four weeks after aspiration sclerotherapy compared to baseline measurements, obtained by ultrasonography. As secondary outcomes, proportional volume reduction, recurrence, symptomatic relief and improvement of health-related quality of life will be assessed. Furthermore, safety and tolerability of the combination of pasireotide and aspiration sclerotherapy will be evaluated.

**Discussion:**

This trial aims to improve efficacy of aspiration sclerotherapy by a combined approach of two treatment modalities. We hypothesize that pasireotide will decrease fluid re-accumulation after aspiration sclerotherapy, leading to effective hepatic cyst reduction and symptomatic relief.

**Trials registration:**

This trial is registered with ClinicalTrials.gov (identifier: NCT02048319; registered on 6 January 2014) and EudraCT (identifier: 2013-003168-29; registered on 16 August 2013).

## Background

Hepatic cysts are fluid-filled cavities originating from congenital malformations of biliary duct cells. Isolated cysts occur at a prevalence of 2.5 to 18%, while polycystic liver disease (PLD) is infrequent [1-4]. Large or multiple hepatic cysts may cause symptomatic disease. Mass-related symptoms include abdominal distension, pain, postprandial fullness, nausea and dyspnea, collectively leading to a compromised health-related quality of life [5,6]. Treatment should be considered in all symptomatic patients [4,7]. Aspiration sclerotherapy (AS) is a minimally invasive procedure indicated for both solitary and dominant hepatic cysts in PLD. AS includes percutaneous drainage of cyst fluid with subsequent intracystic instillation of a sclerosing agent (such as ethanol or tetracycline) aiming to destroy the inner cystic lining of cholangiocytes [8,9]. However, the efficacy of AS is limited due to direct fluid re-accumulation following treatment [10]. Although re-accumulation is transient, strong fluid secretion is associated with lower efficacy rates leading to failure of symptom relief and the necessity to re-intervene [7,10,11].

Somatostatin analogues inhibit cyst fluid production [12]. Several randomized clinical trials demonstrated that somatostatin analogues (octreotide and lanreotide) decrease polycystic liver volume [13-15]. Pasireotide (SOM230) is a more potent somatostatin analogue compared to conventional somatostatin analogues as it has a broader binding profile and stronger affinity to its receptors [16,17]. We hypothesize that pasireotide administration reduces fluid re-accumulation following AS and therefore increases diameter reduction. We expect that inhibited re-accumulation results in effective symptomatic relief and reduced recurrence rates. To this end, we have designed a randomized, double-blind, placebo-controlled clinical trial to evaluate clinical efficacy of AS combined with pasireotide.

## Methods/Design

### Study aim

The primary objective of the SCLEROCYST trial is to evaluate the combined effect of AS and pasireotide on cyst diameter reduction. Secondary objectives are to determine symptomatic relief and cyst recurrence.

### Hypothesis

We hypothesize that administrating pasireotide curtails fluid re-accumulation following AS and thereby increases cyst diameter reduction.

### Study design and setting

**Figure 1:**
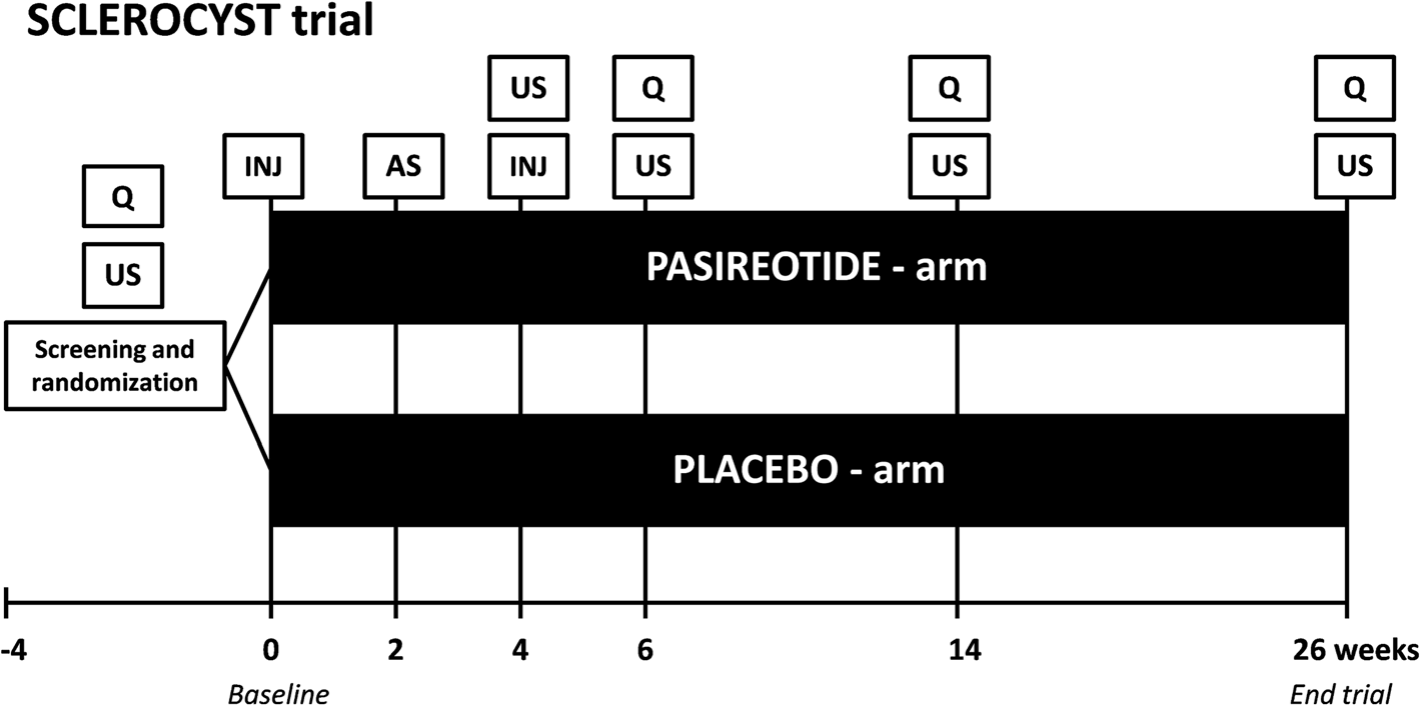
**Study design of the SCLEROCYST trial.** AS, aspiration and sclerotherapy; INJ, injection of pasireotide or placebo; Q, assessment of GIS-, PLD- and SF-36 questionnaires; US, ultrasonography.

The SCLEROCYST trial is a randomized, double-blind, placebo-controlled clinical trial. This single-center study is performed at the Radboud University Medical Center, Nijmegen, the Netherlands. Trial duration will be 30 weeks, consisting of a screening period of four weeks and a treatment period of 26 weeks (Figure 1).

### Randomization and treatment allocation

A total of 34 patients will be randomized in a 1:1 ratio to receive either pasireotide or placebo. All patients will be subjected to AS. Randomization will be performed using block randomization with a block size of four. Included patients will be assigned a randomization number, corresponding to a treatment arm, concealed in a closed envelope. Access to treatment allocation will be restricted to research nurses who prepare and administer the injections. Both patients and treatment allocators will be unaware of the allocated treatment.

### Study population

All patients who are diagnosed with a symptomatic, hepatic cyst that meet the following eligibility criteria will be suitable for participation in this study.

#### Inclusion criteria

The inclusion criteria for this study are as follows:

1. Aged between 18 and 70 years,
2. Indication for treatment with AS, made upon diagnosis of a symptomatic, simple hepatic cyst exceeding 5 cm in diameter,
3. The treated hepatic cyst must be solitary or dominant by nature; a dominant hepatic cyst is the largest cyst surrounded by multiple smaller cysts in patients with PLD and
4. Provision of informed consent to participate.

#### Exclusion criteria

The aspiration sclerotherapy-related exclusion criteria are as follows:

1. Suspicion of a complicated hepatic cyst (active cyst hemorrhage, rupture, or infection),
2. Coagulopathy (international normalized ratio (INR) >2 or platelet count <80 × 10^9^/l) or
3. Severe co-morbidity contraindicating anesthesia (ASA 4 classification).

The pasireotide treatment-related exclusion criteria are as follows:

1. Known long QT syndrome and/or corrected QT interval (QTc) at screening >470 ms;
2. Family history of long QT syndrome or idiopathic sudden death;
3. Uncontrolled or significant cardiac disease including a recent myocardial infarction, congestive heart failure, unstable angina or sustained and/or clinically significant cardiac arrhythmias;
4. Risk factors for torsades de pointes: hypokalemia, hypomagnesemia, hypocalcaemia, clinically significant bradycardia or high grade atrioventricular block;
5. Concomitant disease that could prolong QT interval such as autonomic neuropathy, HIV, cirrhosis, uncontrolled hypothyroidism or cardiac failure;
6. Use of anti-arrhythmic medicinal products or other substances known to lead to QT interval prolongation;
7. Symptomatic cholecystolithiasis;
8. Uncontrolled diabetes, defined as glycated hemoglobin (HbA1C) level >64 mmol/ml despite adequate therapy;
9. Moderate to severe hepatic impairment defined by a Child-Pugh classification of more than six points;
10. History of acute pancreatitis;
11. Hypersensitivity to somatostatin analogues or any component of pasireotide or
12. Non-malignant medical illnesses that are uncontrolled or whose control may be jeopardized by the treatment with the study therapy.

Further exclusion criteria are as follows:

1. Pregnant or nursing women,
2. Use of oral contraception or estrogen supplementation,
3. Intervention (aspiration with or without sclerotherapy or surgical intervention) within six months prior to baseline,
4. Treatment with somatostatin analogues within six months prior to baseline or
5. Any current or prior medical condition that may interfere with the conduct of the study or the evaluation of its results in the opinion of the investigator.

### Trial treatments

#### Aspiration sclerotherapy

All included patients will undergo AS of a single hepatic cyst following standard of care. This single session procedure will be performed by one of our five interventional radiologists, experienced in performing AS. Patients will be admitted the day of AS and will receive intravenous antibiotic prophylaxis (cefazolin 1,000 mg (Eurocept, Ankeveen, the Netherlands) or clindamycin 600 mg (Sandoz, Holzkirchen, Germany) as an alternative in case of allergies). The procedure will be carried out with conscious sedation. Briefly, the cyst will be localized by ultrasonography. Following disinfection and local anaesthesia of the skin, a 5 French pigtail catheter (Cook Medical, Bloomington, United States of America) attached to a three-way tap will be inserted in the hepatic cyst to perform complete fluid evacuation (aspiration). Cyst leakage or communication with vessels or bile ducts will be examined by instillation of contrast medium (Iomeron 300, Bracco Imaging, Konstanz, Germany, up to 20 ml) into the cyst cavity. Contrast medium will then be aspirated from the cyst followed by an injection of 100% ethanol (sclerotherapy). The amount of instilled ethanol consists of 10% of the aspirated cyst volume (up to a maximum of 50 ml). After 10 minutes, ethanol is aspirated and the drain will be removed.

#### Pasireotide

Subjects in the active treatment arm will receive two intragluteal injections of 60 mg pasireotide (SOM230, Novartis, Basel, Switzerland) long-acting release (LAR). The first injection will be administered two weeks prior to AS, the second injection will be administered two weeks following AS. Pasireotide LAR, powder for suspension for injection, is a one-month depot microparticle formulation for intramuscular administration. No dose adjustment is required for race, age, gender, body weight, mild hepatic impairment or renal impairment. The most common adverse events are gastrointestinal by nature and include mild diarrhea, nausea and abdominal pain [18]. These events are more frequent in the first days after treatment initiation and mitigate within time. Moreover, transient hyperglycemia, local injection site skin reactions and headaches are common side effects of pasireotide [18,19]. Previous studies have indicated that pasireotide may prolong the QT interval [20]. Therefore, patients with a prolonged QTc at screening (>470 ms) are not eligible to participate in this study. Known long-term side effects, such as development of gallstones, are not expected in this study given the limited number of injections.

#### Placebo

Patients in the placebo arm will receive two intragluteal injections of 2 ml sodium chloride solution 0.9% (Fresenius Kabi, Hesse, Germany). The first injection will be administered two weeks prior to AS and the second injection will be administered two weeks following AS.

### Study endpoints

#### Primary outcome

The primary outcome of the SCLEROCYST trial is the mean proportional change (%) in cyst diameter of the treated hepatic cyst four weeks after AS, as measured by ultrasound.

#### Secondary outcome

Secondary outcomes are mean proportional change (%) in cyst diameter two, 12 and 24 weeks after AS. Moreover, proportional change (%) in volume, absolute diameter reduction (cm) and rate of cyst recurrence (>80% of original diameter) at follow-up visits will be assessed. In addition, symptoms and health-related quality of life will be measured at baseline, four, 12 and 24 weeks after AS. Finally, any complications or adverse events reported during procedure or follow-up visits will be recorded.

### Ultrasound measurements

The blinded primary investigator will perform cyst diameter measurements by ultrasonography. The treated hepatic cyst will be visualized in two planes to measure each maximal orthogonal diameter (craniocaudal, anteroposterior and mediolateral) three times. As a secondary endpoint, cyst volume will be estimated by multiplying the maximum of each orthogonal diameter by 0.523 [10]. The measurement will be performed using a 3.5 MHz convex transducer (Acuson X150™, Siemens Healthcare, Erlangen, Germany). A blinded independent investigator will repeat all measurements. Both investigators are trained and experienced in measuring hepatic cyst diameter.

### Patient-reported outcome measures

Abdominal symptoms and health-related quality of life will be assessed at screening and during follow-up visits. The standardized gastrointestinal symptom questionnaire (GIS) assesses 11 abdominal symptoms using a seven-point Likert scale ranging from 0 (‘none’) to six (‘severe’) [21]. The Medical Outcomes Study 36-item short-form health survey (SF-36) will be completed to evaluate health-related quality of life [22]. This generic questionnaire assesses health-related quality of life in eight different domain scores and two summarizing (physical and mental) component scores. Additionally, a disease-specific questionnaire for PLD in development (PLD-Q, Radboud University Medical Center, Nijmegen) that assesses frequency and discomfort of 16 PLD-related symptoms over a timeframe of one month, will be completed by all subjects in order to validate this instrument in patients treated for large symptomatic hepatic cysts.

### Study procedures

This study consists of seven visits including one screening visit, three treatment visits and three follow-up visits.

#### Screening visit (within four weeks prior to starting the trial)

Potential subjects with an indication for AS will be asked to participate. Informed consent will be obtained from all participants. Upon providing informed consent, each subject will be screened for eligibility during a separate screening visit performed within 28 days prior to the start of the trial. During this screening visit the following assessments will be made:

1. obtain written informed consent;
2. extensive medical history and physical examination;
3. laboratory markers: potassium, calcium, magnesium, estimated glomerular filtration rate calculated by the Modification of Diet in Renal Diseases (MDRD) formula, platelets, INR, alanine aminotransferase (ALT), aspartate aminotransferase (AST), alkaline phosphatase (ALP), gamma-glutamyl transpeptidase (GGT), total bilirubin (TB), albumin and fasting blood sugar (FBS);
4. urine pregnancy test in female patients with childbearing potential;
5. electrocardiogram: frequency, QTc and atrioventricular conduction;
6. ultrasonography: cyst diameter and volume measurement and
7. questionnaires: GIS-, PLD- and SF-36 questionnaire.

#### First injection (week 0)

The first injection will be administered 14 days prior to AS and indicates the start of this trial. Patients in the treatment arm will receive the first injection of pasireotide LAR 60 mg, while patients in the placebo arm will receive an injection of the sodium chloride solution. The injections will be administered by an independent unblinded health professional who is not involved in the design, execution or analysis of the trial.

#### Aspiration sclerotherapy (week two)

Patients will be admitted the day of AS. Prior to the intervention, adverse events in the previous two weeks will be assessed. Moreover, blood will be drawn to evaluate safety laboratory markers (potassium, magnesium, FBS, ALT, AST, TB and amylase). AS will be performed following standard procedure in both arms. Aspirated cyst fluid will be stored in our laboratory for future analyses.

#### Second injection (week four)

Two weeks following AS (four weeks following the first injection) the patient will visit the outpatient clinic to receive the second injection. Cyst diameter will be assessed. All adverse events will be documented and blood will be obtained. If the following laboratory parameter levels are found, the second injection will not be administered:

1. FBS >9.9 mmol/l;
2. AST or ALT >5 × upper limit of normal (ULN);
3. AST or ALT >3 × ULN with TB >2 × ULN or
4. amylase >10 × ULN.

If the parameters fall within the margin of safety, the second injection will be administered.

#### Follow-up visits (week six, 14 and 26)

Three follow-up visits will be planned at six, 14 and 26 weeks following the first injection (four, 12 and 24 weeks following AS). The researcher will document adverse events and blood will be drawn to assess safety laboratory markers (FBS, ALT, AST, ALP and GGT). The diameter of the treated cyst will be captured and cyst volume will be calculated. The primary endpoint of this study, mean proportional change (%) in cyst diameter at four weeks after AS, will be obtained during the first follow-up visit. Furthermore, abdominal symptoms and health-related quality of life will be measured using the GIS-, PLD- and SF-36 questionnaires.

### Study withdrawal

Subjects can withdraw informed consent and leave the study at any time. The investigator will withdraw a subject from the study for any of the following reasons: pregnancy, consistent failure to adhere to protocol requirements, unacceptable toxicity of study medication, surgical interventions during the study or any other reason if in the best interest of the patient. All data generated up to the time of discontinuation from the study will be analyzed and the reason(s) for discontinuation will be recorded.

### Sample size considerations

Recent data showed that patients treated by AS have a mean diameter reduction of 29.7 ± 19.76% after four weeks [23]. By adding pasireotide, we want to establish a decrease in cyst diameter of 50% after four weeks. A sample size of 15 patients per arm, leading to a total of 30 patients, is required to be able to obtain a significant difference between the two groups (power: 80%; α: 0.05). Taking into account the possibility of 10% protocol violators and/or dropouts, we aim to include 17 subjects per arm, giving a total of 34 patients.

### Statistical analysis

Intention-to-treat (ITT) analyses will be used for all clinical outcome variables. The ITT sample includes all patients who received at least one dose of study medication. In addition, we will carry out a parallel analysis on the per-protocol treated population. This sample is defined as all patients who have received both injections, AS and all ultrasound measurements. The primary outcome of this study is the proportional (%) change in cyst diameter of the treated hepatic cyst four weeks after AS, as measured by ultrasound. The mean diameter of each orthogonal diameter measured by the blinded primary investigator will be used for analysis. Subsequently, we will calculate a mean diameter from the three mean orthogonal diameters. Proportional change will be calculated by dividing the reduction of mean cyst diameter at four weeks by mean cyst diameter at baseline, multiplied by 100. Depending on distribution, values will be presented as mean ± SD or as median ± interquartile range. Consequently, differences between the treatment and placebo arm will be calculated using the independent student’s t-test or the Mann-Whitney U test, respectively. Absolute reduction and volume reduction will be presented and analyzed similar to data for the primary endpoint. Cyst recurrence will be compared between both groups using Fisher’s exact test. The cyst diameter measurements from the second blinded investigator will be used as a control to evaluate intra- and interobserver variability by performing a Bland-Altman analysis. Symptoms of the GIS-questionnaire will be dichotomized (0 to oneversus two to six) and compared between the treatment and placebo arm using the chi-square test. Health-related quality of life domain scores and component scores (SF-36) will be compared between both arms using the Mann-Whitney U test. Frequency tables will be compiled for (serious) adverse events classified according to the standard World Health Organization - Adverse Reaction Terminology (WHO-ART) Body System Dictionary and preferred terms. All statistical analyses will be two-sided, with a critical significance level of 5%.

### Ethical considerations

Ethical approval of the study protocol was given by the Central Committee on Research Involving Human Subjects and by the local accredited Medical Research Ethics Committee of the region Arnhem-Nijmegen, the Netherlands (reference number: 2013/354). This study is to be conducted in accordance with the International Conferences of Harmonization Good Clinical Practice Guidelines, the principles of the Declaration of Helsinki 1964 as modified by the 52nd WMA General Assembly, Edinburgh, Scotland, October 2000 including two notes of clarification on paragraph 29 and 30, and the local national laws governing the conduct of clinical research studies. An independent data and safety monitoring board has been formed to monitor patient safety and treatment efficacy during the trial.

## Discussion

The SCLEROCYST trial is designed to evaluate the additional effect of pasireotide on hepatic cyst diameter reduction in patients treated by AS. We hypothesize that complementary pasireotide treatment results in an augmented treatment efficacy. Multiple studies have shown that AS is an effective and safe method to reduce hepatic cyst volume [7,23,24]. However, cyst reduction is curtailed by fluid re-accumulation following treatment [10]. Fluid secretion by cyst-lining cholangiocytes is mediated by intracellular 3′-5′-cyclic adenosine monophosphate (cAMP). Increased levels of cAMP lead to activation of cystic fibrosis transmembrane conductance regulator (CFTR). Opening of these chloride channels results in chloride efflux, with a subsequent osmotic gradient facilitating water transport into the cyst cavity [17,25]. Somatostatin analogues inhibit cAMP by activating the somatostatin receptor (SSTR), and thereby reduce cholangiocyte fluid production [12,17,26]. Indeed, previous randomized clinical trials showed that both octreotide and lanreotide are capable of reducing cystic liver volume [27].

In the context of this study we want to evaluate whether these inhibitory mechanisms of somatostatin analogues can halt cyst fluid re-accumulation following AS. We have chosen to apply the multi-receptor-targeted, long-acting somatostatin analogue pasireotide (SOM230) [16]. This cyclohexapeptide has a high binding affinity to SSTR 1, 2, 3 and 5 which are all expressed on cholangiocytes [12]. Compared to conventional somatostatin analogues, pasireotide has a broader binding profile and a higher affinity to SSTR 1, 3 and 5. In a polycystic kidney disease rodent model pasireotide decreased intracellular cAMP, cholangiocyte proliferation and expansion of hepatic cysts more effectively than octreotide [17]. Pasireotide is approved in the European Union and United States for the treatment of Cushing’s disease, and is currently being investigated for treatment of other diseases such as acromegaly and neuroendocrine tumors. Two formulations of pasireotide are available: an immediate release and a one-month depot formulation. To ensure adequate pasireotide levels and optimize patient adherence, we have chosen to administer the LAR formulation of pasireotide. A previous pharmacokinetic study of pasireotide LAR in healthy volunteers showed that pasireotide levels reached a peak (*t*_max_) around 20 days following administration [18]. Therefore, to secure adequate levels subsequent to AS, a first injection will be administered two weeks prior to AS, followed by a second injection two weeks following AS.

The strength of this study is its design: a randomized, double-blind, placebo-controlled trial. This will minimize confounding of results. Further, this study is powered on a clinical relevant difference of 50% in the pasireotide arm compared to the expected 30% cyst diameter reduction in the control arm. We believe that a statistical significant finding will have direct implications for future AS treatment. Smaller differences between arms will not be detected in view of power issues. Nevertheless, given the additional costs and potential side effects of pasireotide, we believe that a strong improvement over conventional AS is needed to justify the implementation of this combination treatment. Finally, with this study we hope to achieve a better understanding in the pathological mechanisms behind hepatic cyst development and fluid re-accumulation following AS.

This study has several limitations. First, the effect of treatment will be measured by ultrasonography. Since we will perform cyst measurements within short intervals we have selected ultrasonography as the most appropriate imaging modality to avoid exposure to radiation (from computed tomography scans) and high costs (from magnetic resonance imaging). However, ultrasound measurements are operator-dependent and are therefore susceptible to bias. To minimize biased results, we have added several safety measures. All measurements will be performed according to standard operating procedures and will be executed in triplicate. Moreover, all measurements will be performed by the same investigator who is blinded for treatment allocation. In addition, a second, blinded independent investigator will repeat all measurements. Second, in this study sclerotherapy will be performed with 100% ethanol. Multiple sclerosing agents are in use for AS ranging from ethanol to tetracycline antibiotics, polidocanol, ethanolamine oleate or hypertonic saline [8,28-32]. This might limit the generalization of our findings. On the other hand, ethanol is cheap, widely available and the clinical experience with this agent is large. Finally, AS will be performed by a number of interventional radiologists, theoretically introducing performance bias. Nevertheless, AS is a protocol procedure and large differences in treatment outcome between interventional radiologists are not to be expected.

To summarize, the SCLEROCYST trial is designed to evaluate the additional effect of a somatostatin analogue combined with AS. We aim to reduce fluid re-accumulation following treatment leading to increased cyst diameter reduction and symptomatic relief.

## Trial status

The trial is currently ongoing. Recruitment and inclusion of patients started in April 2014.

## Abbreviations

ALT: Alanine aminotransferase
ALP: Alkaline phosphatase
AS: Aspiration sclerotherapy
AST: Aspartate aminotransferase
cAMP: 3′-5′-cyclic adenosine monophosphate
CFTR: Cystic fibrosis transmembrane conductance regulator
FBS: Fasting blood sugar
GGT: Gamma-glutamyl transpeptidase
GIS: Gastrointestinal symptoms questionnaire
INR: International normalized ratio
LAR: Long-acting release
MDRD: Modification of diet in renal diseases
PLD: Polycystic liver disease
PLD-Q: Polycystic liver disease questionnaire
SF-36: Short form 36 health survey
SSTR: Somatostatin receptor
TB: Total bilirubin
QTc: Corrected QT interval
